# Procalcitonin and C-Reactive Protein for Invasive Bacterial Pneumonia Diagnosis among Children in Mozambique, a Malaria-Endemic Area

**DOI:** 10.1371/journal.pone.0013226

**Published:** 2010-10-14

**Authors:** Núria Díez-Padrisa, Quique Bassat, Sonia Machevo, Llorenç Quintó, Luis Morais, Tacilta Nhampossa, Cristina O'Callaghan-Gordo, Antoni Torres, Pedro L. Alonso, Anna Roca

**Affiliations:** 1 Centre de Recerca en Salut Internacional de Barcelona, Hospital Clínic/Institut d'Investigacions Biomèdiques August Pi i Sunyer, Universitat de Barcelona, Barcelona, Spain; 2 Centro de Investigaçaõ em Saúde da Manhiça, Manhiça, Mozambique; 3 Centro de Investigación Biomédica en Red (CIBER) de Epidemiología y Salud Pública, Barcelona, Spain; 4 Faculdade de Medicina, Universidade Eduardo Mondlane, Maputo, Mozambique; 5 Instituto Nacional de Saúde, Ministerio de Saúde, Maputo, Mozambique; 6 Servei de Pneumologia, Institut Clínic del Tòrax, Hospital Clínic/Institut d'Investigacions Biomèdiques August Pi i Sunyer, Universitat de Barcelona, Barcelona, Spain; 7 Centro de Investigación Biomédica en Red (CIBER) de Enfermedades Respiratorias, Bunyola, Spain; Columbia University, United States of America

## Abstract

**Background:**

Pneumonia is the major cause of mortality and morbidity in children worldwide. Procalcitonin (PCT) and C-reactive protein (CRP) are used in developed countries to differentiate between viral and bacterial causes of pneumonia. Validity of these markers needs to be further explored in Africa.

**Methodology and Principal Findings:**

We assessed the utility of PCT and CRP to differentiate viral from invasive bacterial pneumonia in children <5 years hospitalized with clinical severe pneumonia (CSP) in rural Mozambique, a malaria-endemic area with high HIV prevalence. Prognostic capacity of these markers was also evaluated. Out of 835 children with CSP, 87 fulfilled definition of viral pneumonia and 89 of invasive bacterial pneumonia. In absence of malaria parasites, levels of PCT and CRP were lower in the viral group when compared to the invasive bacterial one (PCT: median = 0.21 versus 8.31 ng/ml, p<0.001; CRP: 18.3 vs. 185.35 mg/l, p<0.001). However, in presence of malaria parasites distribution between clinical groups overlapped (PCT: median = 23.1 vs. 21.75 ng/ml, p = 0.825; CRP: median = 96.8 vs. 217.4 mg/l, p = 0.052). None of the two markers could predict mortality.

**Conclusions:**

Presence of malaria parasites should be taken into consideration, either for clinical or epidemiological purposes, if using PCT or CRP to differentiate viral from invasive bacterial pneumonia in malaria-endemic areas.

## Introduction

Pneumonia is the major cause of morbidity and mortality in children worldwide. More than 90% of the estimated 1.6 million annual pneumonia deaths in children <5 years occur in developing countries [1] and are mainly due to bacteria [2]. However, viruses are isolated in 50–60% of in-hospital pneumonia episodes [3]–[7] and clinical presentation between the two pathogens is poorly specific. Furthermore, laboratory diagnosis to determine viral or bacterial etiology lack sensitivity and their use is limited in developing countries.

In rural Africa, pneumonia diagnosis is mainly based on WHO's Integrated Management of Childhood Illness (IMCI), a set of highly sensitive clinical algorithms developed to guide management of most common mortality causes in children. IMCI has contributed to the reduction of pneumonia childhood mortality [8], [9]. Its algorithms are, however, unable to discriminate between bacterial and non-bacterial causes of pneumonia while only the formers require treatment with antibiotics. Any improvement on clinical endpoints to differentiate bacterial pneumonia with no requirement of laboratory facilities might help management of treatment. The current pneumonia diagnosis also has implications in determining the true burden of pneumonia in epidemiological studies and in measuring the effects of interventions such as vaccination against specific bacteria [10], [11].

Biomarkers to differentiate viral from bacterial infections have been evaluated mostly in the developed world to support clinical diagnosis and to be applied as rapid diagnostic tests (RDT). Despite disparity in results [12], [13], procalcitonin (PCT) and C-reactive protein (CRP) are used for etiological diagnosis in children with pneumonia, assuming higher levels of both markers in bacterial infections when compared to viral ones. Validity of these proxy measures to diagnose pneumonia due to bacteria needs to be assessed in the African continent. *Plasmodium falciparum*, highly prevalent among children in most of Africa, increases levels of CRP [14], [15]. Epidemiological data from a pneumococcal vaccine trial conducted in South Africa, a country with virtually no malaria, suggested that PCT and CRP can contribute to increase pneumonia endpoint specificity resulting in an increase of measured efficacy [10]. However, improvement of specificity was less marked in a similar study conducted in The Gambia, a malaria-endemic country [11]. A recent study in Malawi shows that PCT and CRP in presence of bacterial pneumonia/meningitis were elevated in both HIV-uninfected and infected children [16], but data on malaria in this study were poor. How malaria parasitemia and other prevalent co-morbidities may alter PCT and CRP levels in presence of pneumonia remains to be explored.

In this study, we assessed the utility of PCT and CRP to differentiate viral from invasive bacterial pneumonia in children in Manhiça, a rural malaria-endemic area of Mozambique with high HIV prevalence. Prognostic capacity of these markers was also evaluated.

## Methods

### Study area and population

This study was conducted by the *Centro de Investigação em Saúde da Manhiça* (CISM) at the Manhiça District Hospital (MDH), the referral health facility for Manhiça District, a rural malaria-endemic area of Southern Mozambique with subtropical climate [17].

Since 1996, the CISM has been running continuous Demographic Surveillance System (DSS). The DSS covers 500 km^2^ and approximately 80000 inhabitants, 18% are children <5 years. Under-five mortality rate was 138.6/1000 in 2005 [18]. Each child living within the DSS area is issued a unique permanent identification number allowing tracking of demographic and clinical data collected at the hospital.

Severe pneumonia accounted for 16% of admissions to MDH and 11% of case-fatality rate among children <2 years in 2004–2006; 19% of these children presented clinical malaria [19]. Severe malnutrition prevalence in children <5 years admitted to MDH was 10% in 2001–2003 [20]. HIV prevalence among pregnant woman attending to hospital antenatal clinic was 23.6% in 2004 [21]. Antiretroviral therapy at the time of delivery has been available in MDH since 2003. Mother-to-child transmission rate was estimated to be 12.4% during that period. More than 25% of HIV-infected children died before 1 year [22].

### Hospital surveillance and clinical management

The MDH is a 110 bed hospital with 36 paediatric beds. Since 1997, MDH and CISM have operated round-the-clock surveillance of all paediatric visits [17]. Finger prick blood is obtained for malaria determination and packed-cell volume (PCV) in children with fever (axillary temperature ≥37.5°C) or history of fever in the last 24 hours. Blood cultures are performed upon hospital admission for all children <2 years or older with axillary temperature ≥39°C or other severity signs. Lumbar puncture is performed in hospitalized children with suspicion of sepsis or neurological impairment [23].

### Patient's selection and sample collection

This analysis is part of a larger study designed to describe clinical and epidemiological characteristics of children <5 years admitted to the MDH with suspected clinical severe pneumonia (CSP) between 20^th^ September 2006 and 19^th^ September 2007 [7] (Bassat, Q. et al., unpublished). CSP was defined as cough and difficult breathing with increased respiratory rate according to age group and at least one of the following signs: indrawing, nasal flaring, grunting or crackles. Written informed consent was obtained from all participants parents or legal guardians involved in the study. The study was approved by the Mozambican National Bioethics Committee and the Institutional Review Board of the *Hospital Clínic de Barcelona* (HCB).

As part of the study protocol, blood sample was collected on admission for full blood cell count and PCT/CRP determination. Nasopharyngeal aspirate (NPA) was collected to detect respiratory viruses. Blood culture and malaria determination were extended to all children with CSP. Chest-x-rays were also performed. Children residing in the DSS area were offered voluntary HIV counseling and testing. Additional written informed consent and finger prick blood were required.

According to definitions, children with CSP were classified in two groups, viral and invasive bacterial pneumonia. Children who presented clinical suspicion of *Pneumocystis* pneumonia (PCP) or, according to parents/legal guardian, had received antibiotics before admission were excluded from the analysis.

Asymptomatic healthy children <5 years from the DSS area were randomly selected to participate into this study as part of the control group. After parental/legal guardian consent, blood was collected for determination of malaria and PCT/CRP levels. No other tests were performed to this group.

### Study groups

Viral pneumonia was defined when a child presented on admission with a normal chest x-ray or with abnormalities other than alveolar consolidation or pleural effusion [24], <15×10^9^/l leukocytes in blood, a negative blood culture and at least one of the following viruses isolated from NPA: respiratory syncytial virus (RSV), influenza (Flu), parainfluenza (PIV), human metapneumovirus (hMPV) and adenovirus (ADV).

Invasive bacterial pneumonia was defined when a bacteria was isolated from blood, excluding potential contaminants [*Staphylococcus epidermidis*, *Viridans* group *Streptococci* and *Bacillus* spp. (non *anthracis*)].

Control group was defined as healthy children from the community with absence of fever, signs or symptoms of illness and *P. falciparum* parasites.

### Laboratory methods

*P. falciparum* parasites were detected by microscope observation of thick and thin Giemsa-stained blood films [25]. PCV was measured using microcentrifugue and a Hawksley hematocrit reader card (Hawksley & Sons Ltd, UK). Blood cultures were performed using an automated system (BACTEC® 9050; Becton-Dickinson, Franklin Lake, NJ, USA). Positive blood cultures were examined following standard procedures [20], [26].

To detect RSV, Flu, PIV, hMPV and ADV from NPA two single polymerase chain reactions (PCR) and two multiplex PCRs were performed [7].

HIV testing was done using two RDT: *Determine*® (Abbott Laboratories, North Chicago, IL, USA) and *Unigold*® (Trinity Biotech, Bray, Ireland). HIV-1 infection was confirmed using antigen DNA-PCR Roche *HIV-1 DNA test*® (Roche Molecular Systems, Branchburg, NJ, USA) for <18 months positive children and for those cases with discordant results from the two RDT.

Plasma obtained from 1.5 ml of blood in EDTA (5 minutes, 1500 rpm) after full blood cell count was stored at -20°C until processing at HCB/*Institut d*'*Investigacions Biomèdiques August Pi i Sunyer*. PCT quantification was performed using automated immunoanalysis with *Liaison*® (Diasorin, Saluggia, Italy) or *Kryptor Compact*® (Brahms, Hennigsdorf, Germany). For CRP, immunoturbidimetric assay with *ADVIA Chemistry CRP_2*® (Siemens Medical Solutions Diagnostics, Tarrytown, NY, USA) was used. Limits of detection of these techniques were 0.04 ng/ml, 0.02 ng/ml and 4 mg/l respectively.

### Data management and statistical analysis

Data were double entered using Fox Pro version 2.6 (Microsoft Corporation, Redmond, WA, USA) and analyzed using STATA version 11 (Stata Corporation, College Station, TX, USA). Discrepancies in data entry were resolved by referring to the original forms.

Proportions were compared using Chi-square test. Distributions of both markers were evaluated by mean of Kruskall-Wallis test. Linear regression models were estimated to evaluate the difference of PCT and CRP levels, adjusted by HIV, hematocrit, oxygen saturation and malnutrition. P-value of 0.05 or lower was considered significant.

Optimal cut-offs to determine viral (used as reference) and invasive bacterial pneumonia were investigated using receiver operating characteristic analyses.

## Results

### Study profile

A total of 835 children admitted to MDH during the study period met study entry criteria, 190 (23%) fulfilled definition for viral or invasive bacterial pneumonia. Both plasma and *P. falciparum* determinations were available for 176 (93%) of these children, 87 (49%) corresponding to the viral group and 89 (51%) to the invasive bacterial one. 93 viruses were detected from the 87 children in the viral group (6 viral co-infections), being the most prevalent viruses in decreasing order: ADV (n = 33), RSV (n = 18), hMPV (n = 16), Flu (n = 15), PIV (n = 11). 90 bacteria were detected from the 89 children in the invasive bacterial group (1 bacterial co-infection), being the most prevalent bacteria in decreasing order: *Streptococcus pneumoniae* (n = 42), *Haemophilus influenzae* type b (n = 16), *Staphylococcus aureus* (n = 7), *Escherichia coli* (n = 6), *Salmonella* spp (n = 6), others (n = 13). A total of 37 children met criteria of the control group.

Overall, 11% (17/157) of the children in the clinical groups with outcome data died in-hospital. All fatalities occurred in the invasive bacterial group. Comparison of descriptive and clinical features among groups is shown in Table 1.

**Table 1:** Descriptive and clinical features of children <5 years included in the viral, invasive bacterial and control groups.

Variables	Viralpneumonia(n = 87)	Invasive bacterialpneumonia(n = 89)	Controlgroup(n = 37)	p-value
Age				
<1 month	3 (3)	6 (7)	0 (0)	
1-<12 months	32 (37)	40 (45)	13 (35)	0.223
1-<5 years	52 (60)	43 (48)	24 (65)	
Sex				
Male	57 (66)	55 (62)	24 (65)	0.868
Female	30 (34)	34 (38)	13 (35)	
Malnutrition* (n = 155)				
No	71 (83)	47 (68)	NA†	0.036
Yes	15 (17)	22 (32)	NA	
Hematocrit (n = 175)				
≥33%	47 (54)	31 (35)	NA	
15 -<33%	40 (46)	56 (64)	NA	0.031
<15%	0 (0)	1 (1)	NA	
*P. falciparum*				
No	64 (74)	82 (92)	NA	0.001
Yes	23 (26)	7 (8)	NA	
HIV (n = 110)				
No	58 (95)	23 (47)	NA	<0.001
Yes	3 (5)	26 (53)	NA	
Mortality (n = 157)				
No	85 (100)	55 (76)	NA	<0.001
Yes	0 (0)	17 (24)	NA	
Oxygen saturation‡ (n = 174)				
≥94%	53 (61)	44(50)	NA	
90 - <94%	16 (18)	19 (22)	NA	0.377
<90%	18(21)	24 (28)	NA	

**NOTE.** Data are n (%) of patients, unless otherwise indicated.

*Weight to age z-score <3 SDs from U.S. reference population.

† NA: Not applying. When data from the control group does not apply, the p-value is obtained from the comparison of the two clinical groups.

‡ Measured with pulsioximetry.

### Evaluation of causes attributable to pneumonia

#### PCT values comparing control, viral and invasive bacterial groups

**Figure 1:**
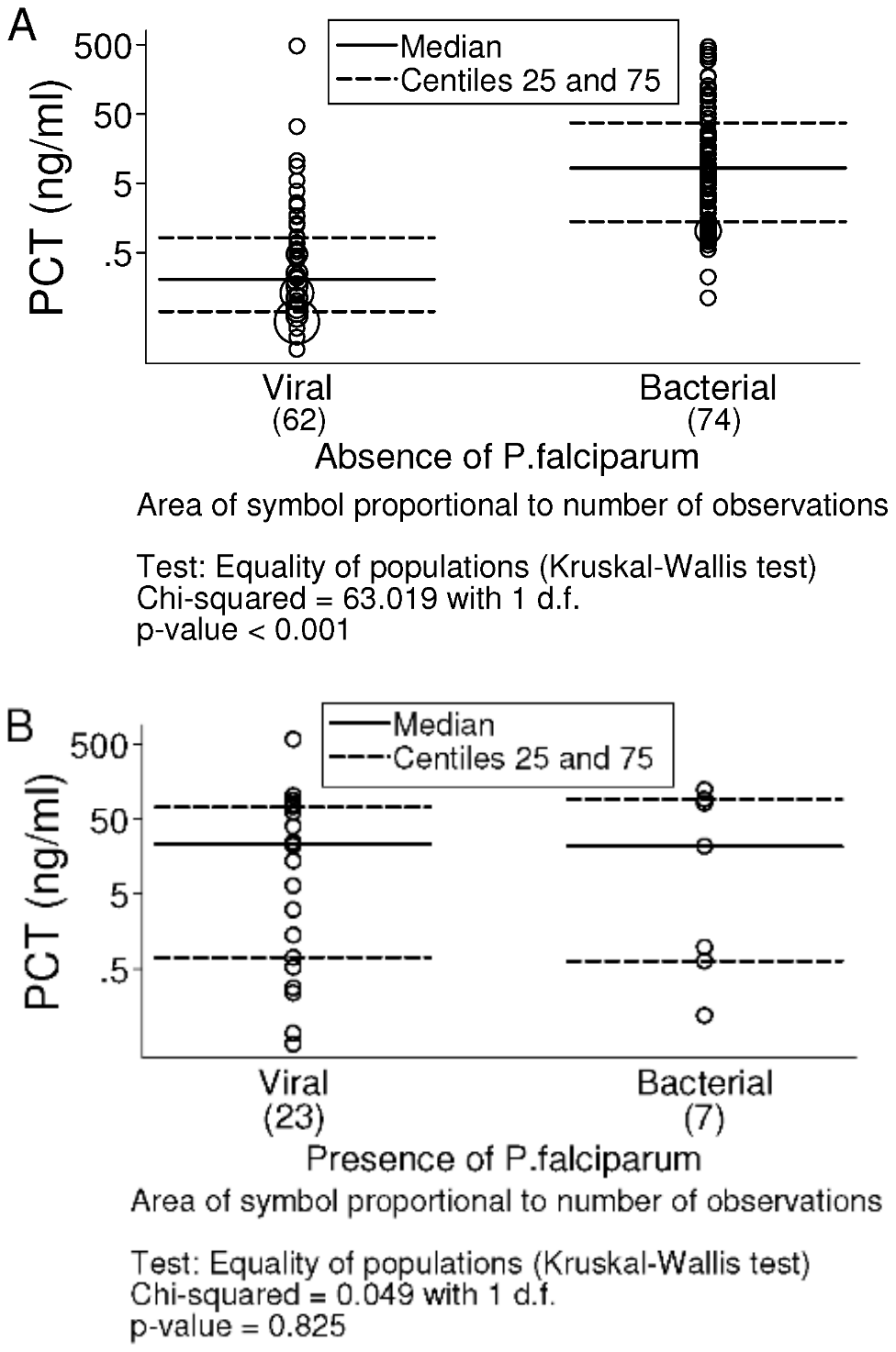
Distribution of procalcitonin (PCT) concentrations in the viral and the invasive bacterial group.

Median PCT concentration in the control group was 0.05 ng/ml. Values in this group were lower compared to clinical groups (n = 37 versus n = 166, p<0.001). In the viral group median values were 0.21 ng/ml and 23.1 ng/ml in absence and in presence of *P. falciparum* (n = 62 and n = 23, p<0.001). In the invasive bacterial group median values corresponded to 8.31 ng/ml and 21.75 ng/ml in absence and in presence of *P. falciparum* (n = 74 and n = 7, p = 0.953). In absence of *P. falciparum* (Figure 1A), PCT distribution between viral and invasive bacterial groups was different (p<0.001) and presented low overlap of values. In presence of *P. falciparum* (Figure 1B), PCT distribution between clinical groups clearly overlapped (p = 0.825).

#### CRP values comparing control, viral and invasive bacterial groups

**Figure 2:**
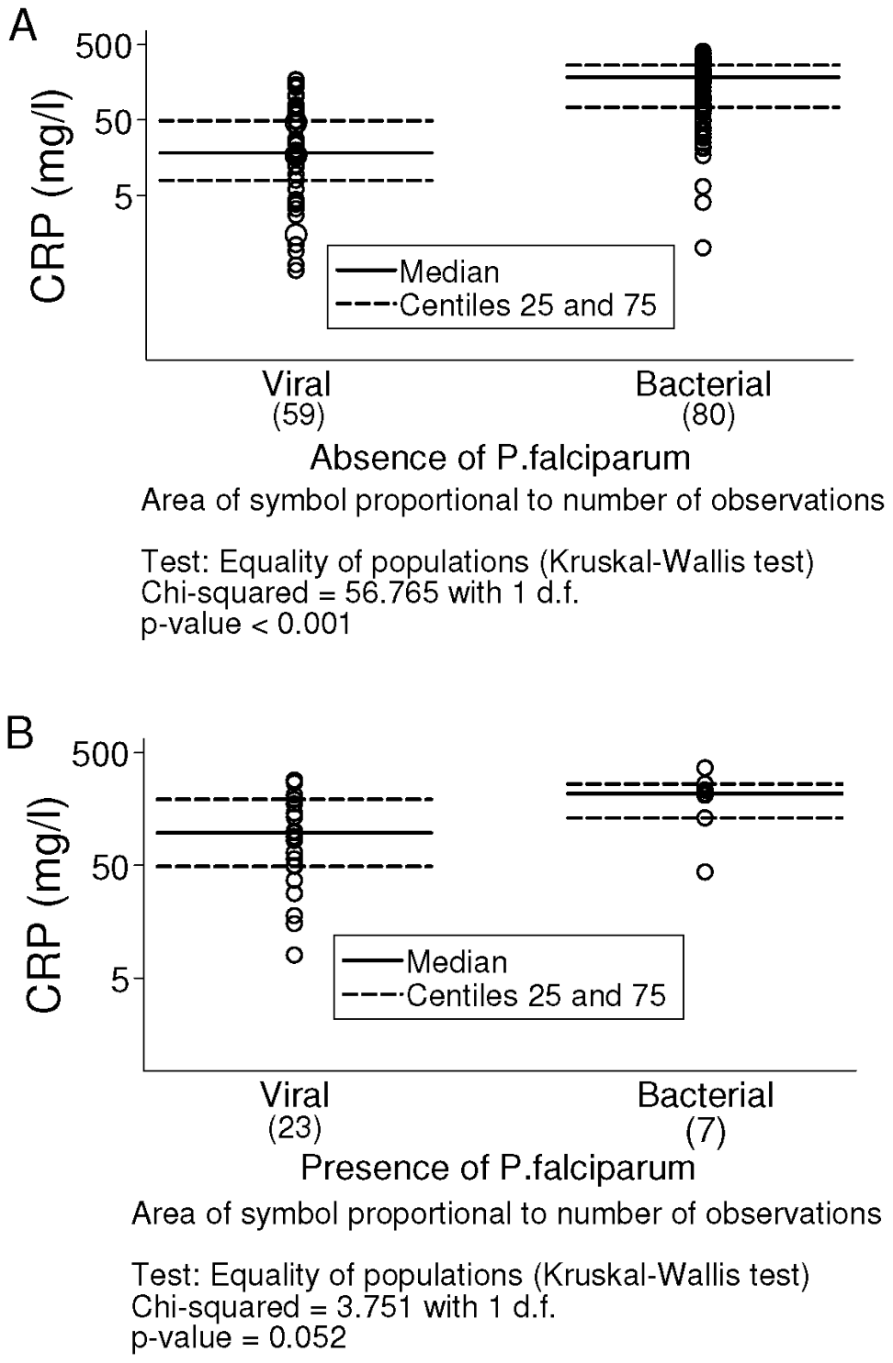
Distribution of C-reactive protein (CRP) concentrations in the viral and the invasive bacterial group.

Median CRP concentration in control group was 4 mg/l. Values in this group were lower compared to clinical groups (n = 33 versus n = 169, p<0.001). In the viral group median values were 18.3 mg/l and 96.8 mg/l in absence and in presence of *P. falciparum* (n = 59 and n = 23, p<0.001). In the invasive bacterial group median values corresponded to 185.4 mg/l and 217.4 mg/l in absence and in presence of *P. falciparum* (n = 80 and n = 7, p = 0.365). In absence of *P. falciparum* (Figure 2A), distribution between viral and invasive bacterial groups was different (p<0.001) and presented low overlap of values. In presence of *P. falciparum* (Figure 2B), CRP distribution between clinical groups overlapped (p = 0.052).

Further analyses were performed in absence of *P. falciparum* due to the observed effect of malaria parasites in PCT and CRP levels.

#### PCT and CRP intra clinical groups variability

PCT and CRP distribution was compared within clinical groups according to virus detected (viral group) or bacteria isolated (invasive bacterial group). In absence of *P. falciparum* no differences were found in PCT and CRP distribution within viruses (p = 0.482 and p = 0.649) (Figure S1, online). The same occurred in PCT and CRP distribution within invasive bacteria (p = 0.145 and p = 0.513) (Figure S2, online).

#### Adjusted analysis of PCT and CRP levels

As shown in Table 2, when adjusting PCT levels with clinical variables in absence of malaria parasites only clinical group (viral versus invasive bacterial) was significantly associated to PCT levels (p<0.001). On the contrary, CRP levels were associated with clinical group and hematocrit (p<0.001 and p = 0.036). None of the both markers was associated to HIV status (p = 0.854 and p = 0.331 respectively).

**Table 2:** Adjusted analysis of markers levels.

Variables	PCT (n = 82)			CRP (n = 81)		
	Proportional difference	95% CI	p-value	Proportional difference	95% CI	p-value
Group						
Viral	1			1		
Invasive bacterial	33.68	10.61–107	<0.001	7.86	3.7–16.69	<0.001
HIV						
No	1			1		
Yes	0.88	0.24–3.31	0.854	0.66	0.28–1.54	0.331
Hematocrit						
≥33%	1			1		
15 -<33%	1.28	0.46–3.59	0.631	2.05	1.05–3.99	0.036
<15%	0.82	0.01–67.85	0.927	1.26	0.07–21.32	0.873
Oxygen saturation						
≥94%	1			1		
90 - <94%	1.51	0.48–4.78	0.475	1.26	0.6–2.68	0.537
<90%	1.13	0.33–3.96	0.842	1.7	0.76–3.8	0.193
Malnutrition						
No	1			1		
Yes	0.77	0.26–2.24	0.622	0.94	0.47–1.92	0.88

#### PCT and CRP diagnostic accuracy

In absence of *P. falciparum*, the area under the curve (AUC) in predicting clinical group was 0.9 for PCT and 0.87 for CRP (n = 129, p = 0.32). PCT and CRP cut-offs were maximized respectively at 0.72 ng/ml (sensitivity 94.6%, specificity 74.2%) and 20.9 mg/l (sensitivity 95%, specificity 54.2%). Combination of both markers did not improve diagnostic profile (data not shown).

### Evaluation of pneumonia prognosis

**Figure 3:**
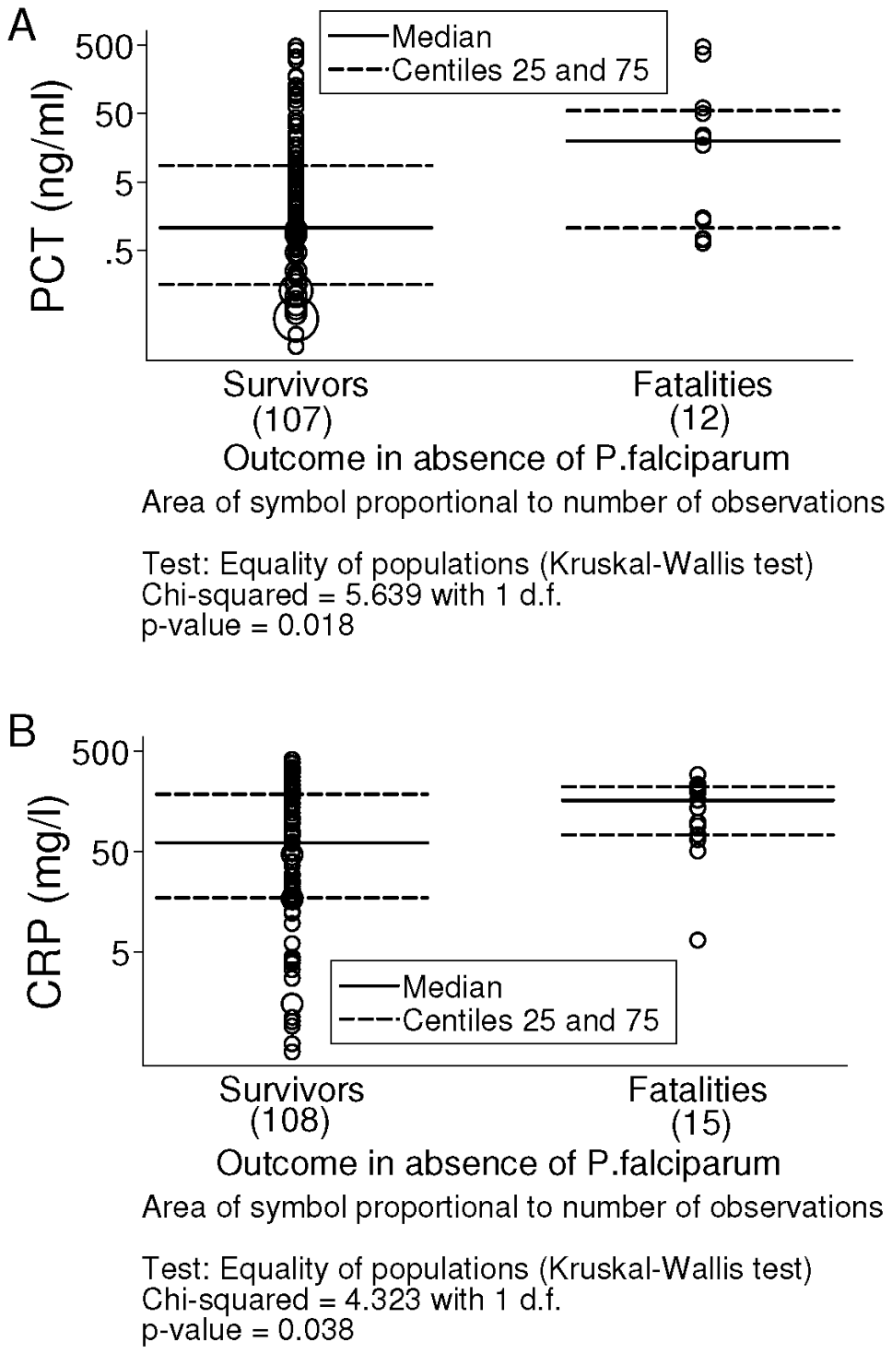
Distribution of procalcitonin (PCT) and C-reactive protein (CRP) concentrations in absence of *P. falciparum* according to outcome.

In absence of *P. falciparum*, PCT distribution between survivors and fatalities was different (n = 107 and n = 12, p = 0.018). However, an important overlap of values was observed between the two groups (Figure 3A). Median values were 1.08 ng/ml and 19.75 ng/ml respectively. For CRP, distribution between outcomes in absence of *P. falciparum* was different (n = 108 and n = 15, p = 0.038). However, an important overlap of values was observed between the two groups (Figure 3B). Median values were 60.95 mg/l and 161.3 mg/l. The AUC for PCT and CRP in predicting death was poor (0.68 and 0.64, n = 113, p = 0.5). When considering only the invasive bacterial group in absence of *P. falciparum*, there was also an important overlap of PCT and CRP values between survivors and fatalities (data not shown).

## Discussion

We have assessed the usefulness of PCT and CRP to differentiate viral from invasive bacterial pneumonia in rural Africa, where malaria, HIV and other co-morbidities are common. Our main finding is that PCT and CRP can not differentiate between these two clinical groups among hospitalized children with malaria parasites. However, both markers can differentiate these two groups in absence of malaria parasites and independently of their HIV status. None of the two markers could predict mortality.

According to our results PCT and CRP have similar distribution among study clinical groups in presence of *P. falciparum*. This contrasts with data from Malawi, where authors showed no significant differences in PCT and CRP levels between children with and without malaria infection [16]. An explanation could be that 74% (280/377) of the cases with signs of pneumonia/meningitis presented with serious bacterial infection and only 4% (13/360) of cases were positive for *P. falciparum.* We do not know the clinical group of these 13 cases. Along with our findings, studies conducted in The Gambia and Tanzania observed that malaria infection increased levels of CRP [14], [15]. Although our results show restrictions on the use of PCT and CRP in a malaria-endemic context, presence of *P. falciparum* can rapidly be assessed with a RDT. In African malaria-endemic countries efforts are being done to establish malaria parasitological diagnosis as routine practice [27].

Strategies to reduce pneumonia burden of disease and severity include prevention through early diagnosis and selection of appropriate treatment. In a context where pneumonia plays an important role in high in-hospital mortality and where diagnostic facilities are poor, all children meeting WHO criteria for CSP should be treated empirically with parenteral antibiotics. Our study suggests that in in-hospital children without WHO criteria for parenteral antibiotics, elevated levels of PCT or CRP in absence of malaria parasites could indicate the need of antibiotics. Additionally, in absence of malaria parasites elevated levels of PCT and CRP could warn of invasive bacterial pneumonia at peripheral health posts and outpatient departments. This might respectively facilitate transfer to hospital and admission, as well as rationalization and prioritization of antibiotics administration.

Standardized definitions of pneumonia in epidemiological studies are still a challenge and measurement of disease burden and efficacy in vaccine trials is necessary using proxy measures. WHO and CDC jointly developed a generic protocol to measure burden of pneumonia using standardized definitions based on radiological interpretation [24]. Although these definitions have been used to determine pneumococcal and *Haemophilus influenzae* pneumonia in epidemiological and intervention studies [28]–[32]; their applicability in rural settings of developing countries is limited, even in research studies, due to the scarcity of radiological facilities. Taking this into account, researchers from South Africa aimed to improve definitions that are easier to apply in the African context. Such definitions included measurement of PCT and/or CRP on admission of children presenting with clinical pneumonia [10]. As we have shown, improvement of epidemiological definitions with PCT and/or CRP may be limited in malaria-endemic countries, as malaria parasites increase levels of both markers independently of the pathogen responsible of pneumonia. This may help explain why values of specificity found in The Gambia [11] were lower than those detected in South Africa [10] when using similar pneumonia definitions, as only the former is a malaria-endemic country. To our understanding, specificity of the definition will be affected by malaria endemicity and seasonality of the study area, but this needs to be further explored.

To determine prognostic markers is another daily challenge for clinicians in resource-limited settings. We also evaluated PCT and CRP as predictors of mortality among clinical groups without *P. falciparum*. Although the study was underpowered for this outcome, no trend was observed. Similarly, PCT and CRP did not demonstrate any value in predicting death among Malawian children with signs of pneumonia or meningitis [16].

The major limitation of this study is related to the definitions of the clinical groups. On one hand, we expect some miss-classifications in the viral group due to undetectable incipient bacterial co-infection [33]. If so, real differences would have been wider than currently observed. Most important, invasive bacterial group represents a reduced spectrum of pneumonias attributable to bacteria. Overall, only 23% of in-hospital children with CSP fulfilled viral or invasive bacterial pneumonia definitions. None of these limitations invalidated our conclusions. However, our results cannot asses the need of antibiotic in non bacteremic cases of pneumonia associated to bacteria. Additionally, how PCT and CRP levels are modified by other prevalent causes of pneumonia in an area with high HIV-prevalence, such as PCP, should be assessed in future studies.

In conclusion, this study provides evidence of the potential utility of PCT and CRP to differentiate viral from invasive bacterial pneumonia in children free of *P. falciparum* in areas with high prevalence of HIV infection. How *P. falciparum* parasites increase PCT and CRP levels independently of the pathogen associated to pneumonia should be considered in the application of these markers for clinical and/or epidemiological purposes.

## Supporting Information

Figure S1 Distribution of procalcitonin (PCT) amd C-reactive proteinin (CRP) concentrations within viruses in absence of *P. falciparum.*
(1.48 MB TIF)

Figure S2 Distribution of procalcitonin (PCT) amd C-reactive proteinin (CRP) concentrations within bacteria in absence of *P. falciparum.*
(1.48 MB TIF)
