# Supplementary figures and images for: Procalcitonin and C-Reactive Protein for Invasive Bacterial Pneumonia Diagnosis among Children in Mozambique, a Malaria-Endemic Area
Source: PLoS One. 2010 Oct 14;5(10):e13226. doi: 10.1371/journal.pone.0013226 (PMC2954814; doi:10.1371/journal.pone.0013226)

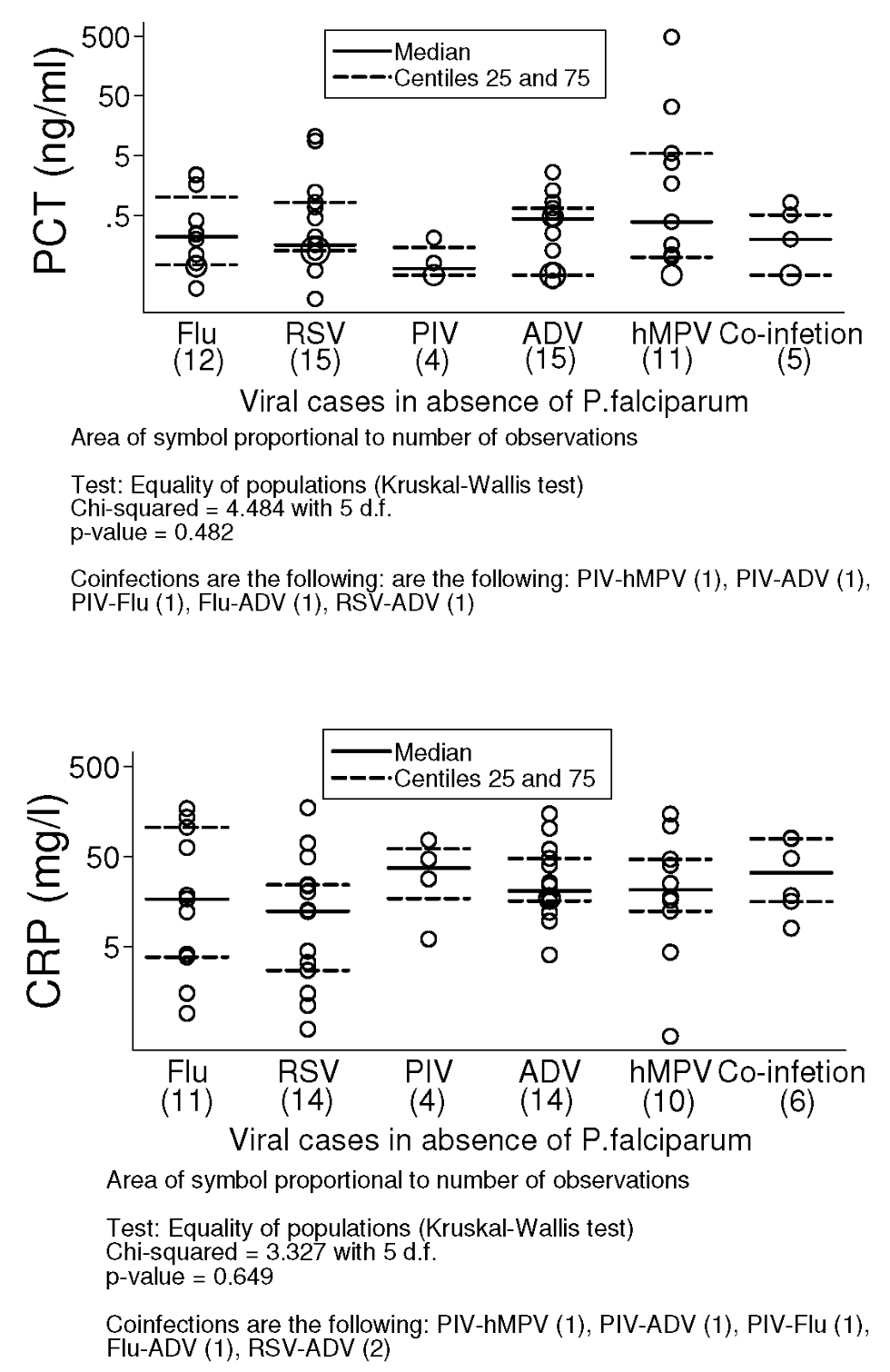

Supplement: Figure S1 — Distribution of procalcitonin (PCT) amd C-reactive proteinin (CRP) concentrations within viruses in absence of P. falciparum. (1.48 MB TIF) [file pone.0013226.s001.tif]

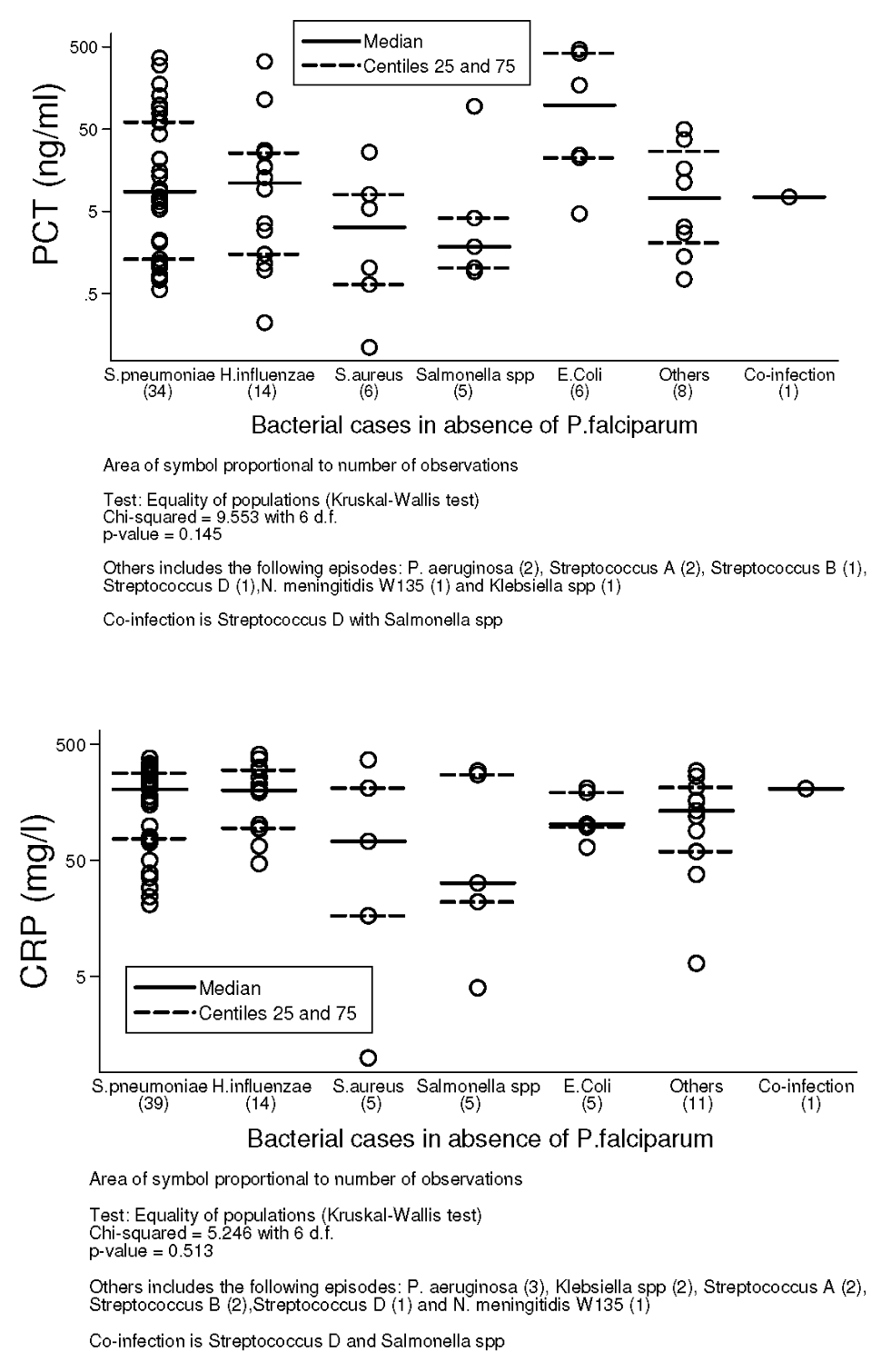

Supplement: Figure S2 — Distribution of procalcitonin (PCT) amd C-reactive proteinin (CRP) concentrations within bacteria in absence of P. falciparum. (1.48 MB TIF) [file pone.0013226.s002.tif]
